# A scientific communication mentoring intervention benefits diverse mentees with language variety related discomfort

**DOI:** 10.58734/plc-2023-0020

**Published:** 2023-10-04

**Authors:** Carrie A. Cameron, Hwa Young Lee, Cheryl B. Anderson, Erin K. Dahlstrom, Shine Chang

**Affiliations:** 1 Behavioral Science, The University of Texas MD Anderson Cancer Center, United States; 2 Cancer Prevention Research Training Program, The University of Texas MD Anderson Cancer Center, United States

**Keywords:** research training, impostor, diversity, equity and inclusion, dialect, mentoring, scientific communication, language variety, belonging

## Abstract

We studied social-psychological effects over time of a faculty-mentor workshop intervention that addressed attitudes associated with language variety and their impact on scientific communication (SC) skill development of PhD and postdoctoral STEM research trainees (*N* = 274). Six months after their mentors attended the workshop, all mentees had significant gains in productivity in speaking tasks. In particular, mentees with high language discomfort rated their quality of communication with their mentor and their enthusiasm about communicating more highly (*p* < .05 for both measures), compared to mentees with low language discomfort. In addition, mentees raised speaking nonstandardized varieties of English reported significant reductions in discomfort related to language use (*p* = .003), compared to mentees raised speaking standardized English. We conclude that training mentors to understand and respond to language diversity and development results in multiple beneficial outcomes for mentees, including the amelioration of language-variety related discomfort in the research environment.

Language and its many components, for instance, accent, intonation, grammar, vocabulary, register, and conversational style, are potent markers of identity and belonging. Labov’s (1966) landmark study *The Social Stratification of English in New York City*, together with seminal works by Wolfram (1969), Rickford (1980), Smitherman (1986), and others sparked a flourishing of scholarship which sought to capture, describe, define, explain, and apply the dynamics of language variety and language use in society. The relationship of language variety (dialect, sociolect, etc.) and use to identity, especially group identity, sense of belonging, and related constructs, has been extensively studied (Alim & Smitherman, 2012; Bucholtz & Hall, 2005; Dannenberg & Wolfram, 1998; Eckert & Rickford, 2001; Edwards, 2009; Llamas & Watt, 2009; Riley, 2007). Language variety has been associated with stigma, bias, and discrimination (Baugh, 2016; Charity Hudley & Mallinson, 2016; Freynet & Clément, 2019; Gluszek & Dovidio, 2010; Lev-Ari & Keysar, 2010; Lippi-Green, 1997). However, despite the efforts of scholars of sociolinguistics to raise awareness, stigma and bias surrounding the use of nonstandardized language varieties persists in the United States.

In higher education settings, scholars such as Hudley et al. (2020), Dunstan et al. (2015), and Scott & Brown (2008) have highlighted the persistence of linguistic bias among faculty and students. As students advance to the postgraduate education and training environment, these issues may be compounded. For research trainees raised speaking nonstandardized varieties of English (whether as a first language [L1] or an additional one [L2]), stigma, impostor feelings, and experience with bias may be exacerbated by the pressure to acquire the register of writing and speaking deemed acceptable by disciplinary gatekeepers (Aitchison & Lee, 2006; Belcher, 2007; Flowerdew & Wang, 2015) to enter the research community of practice (Lave & Wenger, 1991). The research mentors training their mentees to speak and write professionally have a major, direct influence on the mentees’ progress in learning these skills, which are explicitly associated with their potential for success in academic research. Pressure to publish in peer-reviewed journals and present at professional conferences can be intense. Within the science, technology, engineering, and mathematics (STEM) research training context, proficient scientific communication (SC) can be considered the ultimate currency of career success (including the potential of obtaining extramural funding). For this reason, it is critical that STEM researchers “know the rules” of this register, even though they may not articulate or address this need explicitly but instead sense it and react to it implicitly (Cameron et al., 2013). Within this hyper-prescriptive environment, expressed and unexpressed associations of variety markers (e.g., accent, grammar, lexical choice, and style) with perceived intelligence, competence, and belonging may reside outside of individuals’ awareness, and therefore, remain unexamined. Thus, building awareness among STEM gatekeepers at the postgraduate education and training levels, such as mentors, grant and fellowship reviewers, and journal editors, is an important part of making progress in diversifying the STEM research workforce.

## Diversity, Equity, and Inclusion (DEI) in Federally Funded STEM Research

Underrepresentation of racial/ethnic minoritized (URM) groups in the US STEM research workforce is a persistent concern of academia and of funding agencies. The racial/ethnic categories that are currently recognized as underrepresented in STEM by the US National Institutes of Health (NIH) and National Science Foundation (NSF) are Black or African American; Hispanic or Latino; and American Indian or Alaska Native, Native Hawaiian, and other Pacific Islander (National Institutes of Health, 2022; as of 2022, Asian Americans are not formally recognized as underrepresented in STEM research careers.) While progress has been made, the participation rate of URM groups in the STEM workforce of 13.3% remains lower than their overall population representation, 28.1% (National Science Board, 2020). To broaden participation and promote greater inclusivity, the NIH and the NSF have made significant investments over the last two decades in studying the causes of underrepresentation and solutions for broadening participation in undergraduate and postgraduate STEM disciplines. This work includes supported research experiences such as diversity-focused internships and fellowship programs (Taylor et al., 2017; Unguez et al., 2021); enrichment and social support programs (Lee et al., 2015); study of social-psychological factors, including science identity (Estrada et al., 2011; Stewart, 2022); the sense of belonging and engagement of underrepresented groups from undergraduate to postdoctoral levels (Clements et al., 2022; Estrada et al., 2016; Hernandez et al., 2021); and mentor training that includes DEI content (Dukes et al., 2022; Fadeyi et al., 2020; Pfund et al., 2016; Thakore et al., 2014).

## Development of Scientific Communication Skills in the Academic Research Environment

Although postgraduate SC skills and language variety, especially L2, have been addressed by scholars of sociolinguistics, communication, and language learning (Cargill & O’Connor, 2006; Flowerdew, 2000; Kranov, 2009; Li et al., 2019; Swales, 1990), their relationships to identity and career development have been underappreciated within the STEM community itself, despite the widely acknowledged fundamental importance of communication skills for career progression and retention. The process of developing the scientific register of speaking and writing may be taken for granted, and therefore, not mentored sufficiently, causing stress for both mentors and mentees (Aitchison et al., 2012; Cameron et al., 2013; Kranov, 2009). However, these concerns go beyond the instrumental necessity of writing and publishing theses and articles and presenting research at scholarly conferences. The distance between the mentee’s home language variety on the one hand and the prescribed SC style used in STEM research on the other raises two questions. First, whether mentees experience stereotype threat or impostor feelings because of the way they talk and write, and second, whether mentors carry implicit or explicit bias about their mentees’ suitability for research careers for the same reason. If acquiring the scientific “variety” of English creates barriers to mentees’ progress and socialization, their enthusiasm for remaining in research careers may be diminished. This concern is especially relevant to the mentor-mentee relationship.

## Mentee and Mentor Voices

The relationship between mastering SC and research trainees’ sense of belonging and desire to persist as bona fide STEM investigators is exemplified in the following quotes from native-English or bilingual speakers collected in earlier focus groups and interviews:

[P]articularly now as a doctoral student, I feel that people are ‘smarter’ than I am or have access to a broader range of vocabulary than I have, particularly around scientific conversations—just sort of ways of talking that transfer easier for them to the writing of grants and writing of manuscripts.… [I felt] like I was sort of out of my league.… It’s really been about trying to learn to speak the way they do, to write the way they do, to act the way they do. —First-generation rural African American doctoral student training at an elite, research-intensive urban institution; focus group participant (personal communication, 2018). [I] was considering a faculty position; I’m reconsidering academia now based on thoughts of my ability in scientific communication. —First-generation Spanish-English bilingual postdoctoral fellow training at a prestigious academic medical center; interview participant (Cameron et al., 2013, p. 1502).

The quotes above underscore the unique role of the relationship between language background and communication skills in influencing research career intentions, apart from scientific or technical competence or potential. L2 trainees also express concerns related to language use. A survey of 38 postdoctoral fellows conducted at a major US academic medical center (Cameron et al., 2011) found that 83% believed the way they spoke English had interfered “a lot” or “some” with their professional opportunities, including access to the PI of their lab, not being able to participate in scientific discussions, and delays in hiring and promotion. Barriers cited included “ideas not being accepted or being stolen,” “lack of invitations to collaborate,” “[not] make friends,” and “not talking and asking questions in fear of sounding awkward.” Additional comments included (p. 73):

I couldn’t say my comments, I couldn’t ask questions, so it means that you do not know anything.

I felt I was put aside and the radiation oncologist explained the idea instead of me to my PI.

Similar to the L1 trainees’ quotes, these comments reflect L2 trainees’ deep concerns about the impact of their SC skills on their current progress and future success. The L2 speakers’ responses reflect a sense of alienation and even contempt from their L1 colleagues. This may reflect contextual issues: questions posed in a way that invited direct comparison with participants’ past experiences as L1s in their home environments, participants’ knowledge that they had chosen to work in an English-language environment, or the fact that many participants were seasoned researchers forced to take trainee positions in order to remain in academic research. Regardless of the reasons for their responses, it is clear that these speakers felt disadvantaged in the research environment based on their linguistic skills, not their research skills, and that the disadvantage has implications for career progression.

## Role of Scientific Communication Skills in Research Training and Careers

A 2020 study of biomedical doctoral and postdoctoral trainees (Cameron et al., 2020) found that a hypothesized model fit the data well, indicating that intention to persist in research careers is predicted by SC variables, such as SC productivity (frequency of presenting and writing in the research environment), SC outcome expectations (“What will happen if I work at mastering SC?”), SC self-efficacy, science identity (Chemers et al., 2011), and mentees’ perceptions of the mentoring skills they were receiving for SC directly and indirectly. A previous study revealed that nonstandardized home language variety (“the variety of English I spoke at home while growing up”) was associated with language-related discomfort in the research environment, and that home language variety was more likely to influence discomfort scores than was race/ethnicity or first-generation college student status (Trachtenberg et al., 2018). Mentees who reported being raised with a nonstandardized variety of English or another language were not asked whether they used the nonstandardized variety in the research environment currently, only whether they were raised speaking a variety different than that generally used in the research environment (Trachtenberg et al., 2018). Based on these findings, we designed and launched an interventions-research project to test whether a faculty training workshop could teach mentors skills for influencing the SC-related outcomes of their diverse doctoral and postdoctoral mentees.

## The SCOARE Study

The Scientific Communication Advances Research Excellence (SCOARE) study (NIH R25 GM125640, Cameron C. and Chang S., MPIs) recruited faculty mentors as participants, and mentors nominated their STEM postgraduate mentees to participate in the study concurrently. Both mentors and mentees completed a pre-survey, after which the mentors attended the workshop. Six months after the workshop, mentors and mentees completed a post-survey.

The SCOARE research mentor-training workshop (Dahlstrom et al., 2022) comprises 6 contact hours covering the following topics (see Appendix 1 for the full workshop agenda):

1. Evidence base for the role of SC in increasing intention to remain in research careers;
2. Role of language use in individual and social identity, stereotype threat and impostorism, bias (unconscious or conscious), and discrimination;
3. How language, including register, is acquired and used; how stimulating use of the three modes of writing, presenting (as in rehearsed speaking), and spontaneous speaking (as in lab meetings, conversations with colleagues, etc.) helps mentees develop;
4. Ways to mentor SC authoritatively (scaffolding mentee SC skill development, using acknowledgement frequently, providing quality feedback, providing structure and accountability);
5. How and why to increase and facilitate mentee engagement in the three modes of writing, speaking, and presenting.

The relationship of language variety to identity, stereotype threat, and bias (Topic 2), is the section of the SCOARE intervention intended to help mentors understand language variety and gain strategies to reduce their mentees’ language-related discomfort. Here, we present results from this component of the study, for mentees who were raised speaking nonstandardized varieties of English (including L2) and who reported experiencing discomfort in the research environment related to the way they speak English. Building on our earlier investigation of discomfort attributed to language variety, discomfort was operationalized as two items assessing self-reported feelings of being uncomfortable at work or school and of being judged unfavorably by others because of the way one speaks English. Language discomfort is partially congruent with Gluszek and Dovidio’s (2010a, 2010b) construct of stigma for nonnative accents, but includes language varieties of native speakers as well as of nonnative speakers. Language-related discomfort in the academic research environment has been shown to be more prevalent among speakers of nonstandardized varieties of English, including regional dialect speakers, ethnic sociolect speakers, and L2 speakers, which frequently but not always align with ethnicity and socioeconomic status (Trachtenberg et al., 2018).

### SCOARE: Intervention Phase

Here, we aimed to answer two research questions:

1. Do mentees reporting high language-related discomfort experience different effects than those reporting low discomfort after their mentors attend a SCOARE workshop? If so, in what ways?
2. Does the mentees language-related discomfort change after their mentors participate in a SCOARE workshop? If so, does the group reporting standardized English or the group reporting nonstandardized English experience a greater change?

## Methods

### Participants and Procedure

Participants were doctoral and postdoctoral mentees in biomedical and behavioral sciences nominated by their mentors who attended the SCOARE workshop. To recruit mentors for the workshop, we recruited faculty at major universities and academic biomedical institutions in the United States, including Georgia State University, the University of Colorado Boulder, the University of Chicago, all Texas Medical Center institutions affiliated with the Gulf Coast Consortia, and the University of California, San Francisco. Once mentors completed online consent and agreed to participate in our research study, we asked them to nominate mentees to also participate in the survey. Mentees did not attend the workshop. Two hundred mentors nominated, on average, two mentees each. Of the nominated mentees, 354 of 400 invited nominees (88%) accepted, completed online consent, and met the eligibility criteria (18 years or older and doctoral or postdoctoral fellows with research as their primary focus). The mentee study participants were asked to complete the online pre-workshop survey two weeks before their mentors attended the workshop and the online post-workshop survey six months after their mentors attended the workshop.

The study was approved by The University of Texas MD Anderson Cancer Center’s Institutional Review Board (Protocol 2018-0206). Participants completed the online surveys from 2018 to 2021. The surveys took 10-15 minutes, and participants received a $20 gift card as compensation for each survey.

### Online Instrument

#### Language Variety

Mentees were asked whether they claimed English as a primary language, were bilingual, or claimed another language than English, and if bilingual or L2, were asked to specify the language. (“Primary” was the term most readily understood by our testers to indicate the language they were most fluent in or comfortable with.) They were then asked whether the language they were raised speaking is different from the language they use in the research environment:

“Many US Americans grow up speaking a variety of English that is different from what is usually spoken in the academic environment or on news channels. These varieties are often referred to as ‘dialects.’ Dialects can differ from the ‘standard’ variety by pronunciation, certain grammar rules, vocabulary, style of conversing, and other aspects. Did you grow up speaking one of the following regional/social varieties of English?”

Our purpose in this study was to measure linguistic discomfort in the research environment associated with general sociolinguistic groups that may be associated with stigma and discrimination, rather than to pinpoint specific language varieties in the study population. Data on specific varieties are not reported here due to low numbers. See Appendix 2 for the complete list of language varieties presented.

#### Discomfort

Two discomfort items were developed from prior qualitative work in which participants expressed these sentiments (Cameron et al., 2013). The item “Do you feel that you have been judged unfavorably because of the way you speak English?” was rated with a five-point Likert-type scale using 1 (*definitely not*), 2 (*probably not*), 3 (*possibly*), 4 (*probably yes*), and 5 (*definitely yes*). The item “How often do you feel uncomfortable at work or school because of the way you speak English?” was rated with a five-point Likert-type scale using 1 (*never*), 2 (*rarely*), 3 (*sometimes*), 4 (*often*), and 5 (*always*). Previous studies validated these two items’ face validity and content validity (Cameron et. al., 2020; Trachtenberg et. al., 2018).

Discomfort was not queried in the post-workshop survey in the 2018-2019 cohorts. These items were added to the post-workshop survey beginning in 2020 to examine whether mentees’ discomfort levels changed over time.

#### Quality of Communication

Mentees were asked to rate the quality of their communication with their mentor using a five-point Likert-type scale ranging from 1 (*very low*) to 5 (*very high*) in both pre- and post-workshop surveys.

#### Productivity in Speaking Tasks

Mentees were asked to evaluate how often they had spoken up and made comments in discussions over the previous six months. Responses were rated with a seven-point Likert-type scale ranging from 1 (*never*) to 7 (*every day*). This item was included in both pre- and post-workshop surveys.

#### Impact of Mentors on Speaking Tasks

Mentees were asked to evaluate the degree of influence their primary mentor has had on their productivity or progress in informal speaking about science, for example, speaking up, participating in discussions. Responses were rated with a five-point Likert-type scale using 1 (*strongly decrease*), 2 (*decrease*), 3 (*no influence*), 4 (*increase*), and 5 (*strongly increase*). This item was included in both pre- and post-workshop surveys.

#### Emotion in Speaking Tasks

The Positive and Negative Affect Schedule (PANAS; Watson et al., 1988) was used to assess feelings and emotions regarding scientific speaking. For brevity, we selected four positive (interested, strong, enthusiastic, determined) and two negative items (irritable, nervous) from the PANAS. We asked the mentees to indicate how they felt “right now” about scientific speaking (e.g., giving oral presentations, speaking up in discussions, asking questions). Responses were rated with a five-point Likert-type scale ranging from 1 (*very slightly or not at all*) to 5 (*extremely*). The selected affect items were included in both pre- and post-workshop surveys.

#### Reconsideration of Career Intention due to Writing and Speaking Required

We asked the mentees to rate their agreement with the statement “Thinking about the writing and speaking necessary to be successful makes me reconsider my goal of pursuing a research career,” using a five-point Likert-type scale ranging from 1 (*strongly disagree*) to 5 (*strongly agree*). This item was included in both pre- and post-workshop surveys.

### Analysis

#### Cramér’s V Analysis

To answer the first research question, a Cramér’s *V* analysis was used to assess how strongly the level of discomfort in speaking academic English was associated with language variety. This analysis is useful to determine how strongly two categorical variables are associated (Cramér, 1946).

#### Mixed-Effects Models

To answer both research questions, mixed-effects linear models with the intercept as a random effect were used to assess whether SC outcomes change over time when time (pre- and six-months-post-workshop surveys) is nested within mentee individuals and mentees are nested within mentors. Mixed-effects models are useful to analyze data that are nested or hierarchical in structure, and when used with longitudinal data, they allow for correlations between repeated measurements within each participant to be determined (Detry & Ma, 2016). The models were estimated using maximum likelihood via PROC MIXED in SAS (v. 8.2). Using linear mixed-effects models, we examined whether SC outcomes of mentees changed between pre- and six-months-post-workshop surveys and whether the change depended on the level of language discomfort. The nine SC outcomes included impact of the mentor on speaking tasks, productivity of speaking tasks, quality of communication, and six emotions related to speaking tasks (four positive: interested, enthusiastic, strong, determined; two negative: irritable, nervous). For the purpose of statistical analysis and ease of interpretation, the two discomfort questions were categorized into two categories. If the participants chose response option 3 or higher (possibly, probably yes, definitely yes for judged unfavorably; sometimes, often, always for feeling discomfort at work or school), they were put in the high-discomfort group, while a response option of 2 or lower (probably not, definitely not; rarely, never) determined the low-discomfort group.

In addition, we conducted another series of linear mixed-effect models to examine whether mentee discomfort levels changed over time and if the level of change differed by language variety. For ease of interpretation, “language variety” was hand-coded into two categories: nonstandardized English-speaking (including ethnic sociolect, regional dialect, and L2) and standardized English-speaking (those who reported that their language variety was not different from that spoken in the academic research environment).

## Results

Of the 354 mentees who expressed initial interest to participate in our study, 66 were mentees of mentors who did not attend the workshop. These mentees were removed from the analysis as it falls outside the scope of this study, and the sample size was insufficient to perform a mixed-effects model to compare the groups of interest. Of the resulting 288 mentees, 14 completed only the demographic section (or a very small portion of the pre-workshop survey), and so, were excluded from the analysis. Of the 274 remaining mentees, 238 completed both surveys (retention rate = 88%). In about 60% of cases, two or more mentees of the same mentor participated and completed at least the pre-workshop survey. The remaining 40% were single mentees.

Sample characteristics are shown in Table 1. Most participants were doctoral mentees (73%). Women comprised 65% of the sample, and participants self-identified as members of the following ethnic groups: African American (7%), Asian or Asian American (28%), White (57%), and Hispanic/Latino (12%). More than half identified as native speakers of English (58%), 23% as bilingual speakers, and about 33% as first-generation college students. Approximately half of the participants were pursuing degrees related to basic science (55%), 24% in clinical science, 7% in population science, and 15% in other fields.

### Sample Sizes of Respondents Reporting Varieties Not Spoken in the Research Environment

Table 2 shows the binary categorization for the two discomfort questions. For language variety, 37 mentees (14%) reported that they were raised speaking an ethnic sociolect, 28 (10%) with a regional dialect, and 75 (27%) with English as a second language. Twelve trainees (4%) chose the “Other” response option and reported varieties such as “Nigerian English,” “Farsi,” “Rocky Mt dialect,” “French-inlluenced,” or “Indian/rural.” These 12 trainees were not included in further statistical analysis due to the small group size and the difficulty of putting these diverse responses into one category All mentees not selecting a regional or ethnic variety or L2 were categorized as “standardized” English speakers. Regarding “being judged unfavorably because of the way I speak,” 78 of 274 (28%) mentees belonged to the high-discomfort group and 196 mentees (72%) belonged to the low-discomfort group. Similarly, for “discomfort at work or school because of way I speak,” 70 mentees (26%) belonged to the high-discomfort group and 204 mentees (74%) belonged to the low-discomfort group. There was no significant association between discomfort levels and gender (data not shown). Cramér’s V correlation coefficient for the variable of home language variety and “being judged unfavorably” was .49 (*p* < .001), indicating that mentees perceived being judged unfavorably by home language variety In particular, only 5% of the standardized English–speaking mentees reported feeling judged unfavorably, but 30% of ethnic sociolect speakers, 64% of regional dialect speakers, and 48% of L2 speakers reported that they felt judged unfavorably This pattern was similar but more pronounced for responses to the question about “discomfort at work” related to language variety (Cramér’s *V* correlation coefficient = .56, *p* < .001). Substantial proportions of ethnic sociolect speakers (40%) and L2 speakers (60%), but fewer standardized English–speaking mentees (5%), belonged to the category of high “discomfort at work.” However, responses patterned somewhat differently for regional sociolect speakers, who were less likely to report discomfort at work (7% compared to 64% for the “judged unfavorably” measure), but still more likely than standardized English speakers to do so.

Table 3 shows estimated means (standard errors), *F* statistics (*p* values), and likelihood-ratio R^2^ to compare pre- and six-months-post-workshop survey scores for each SC outcome by the discomfort questions.

### Research Question 1

#### Quality of Communication

Mentees with a high level of “being judged unfavorably” on the pre-workshop survey reported improvement in the quality of communication with their mentors after the mentors attended the workshop (*F* = 8.93, *p* = .003). Those with low levels of discomfort did not report changes in their perception of the quality of communication with mentors (*F* = .30, *p* = .58). Regarding “discomfort at work,” mentee perceptions of the quality of communication did not significantly change between pre- and post-workshop surveys. However, there was a marginal positive change for the high discomfort at work group (*p* = .05).

#### Speaking Productivity

Mentees showed increased productivity in speaking spontaneously at six months post-workshop regardless of their level of “being judged unfavorably” or the level of “discomfort at work” (high discomfort: *F*_judged_unfav_ = 7.41, *p* = .007; *F*_discomfort_at_work_ = 4.36, *p* = .038; low discomfort: *F*_judged_unfav_ = 14.51, *p* < .001; *F*_discomfort_ at_work_ = 17.28, *p* < .001).

#### Impact of Mentors on Speaking Tasks

Mentees with low levels on both discomfort questions reported that their mentors’ impact on encouraging their speaking had increased at six months after the workshop (*F*_judged_unfav_ = 6.41, *p* = .012; *F*_discomfort_at_work_ = 5.95, *p* = .016). However, the scores of mentees with high discomfort did not significantly change for either question, possibly because their scores were relatively high at baseline.

#### Positive Emotions

##### Enthusiastic.

In speaking tasks, the group that expressed high discomfort on both discomfort items was more enthusiastic about speaking post-workshop (*F*_judged_unfav_ = 4.95, *p* = .027; *F*_discomfort_at_work_ = 4.37, *p* = .038). The group expressing low discomfort for both items did not significantly change in enthusiasm over time, although marginal positive change was found (*F* = 3.70, *p* = .056).

##### Strong.

All groups either increased or maintained feeling strong about speaking after their mentors attended the workshops, although a divergent pattern for the two items was identified. For the “being judged unfavorably” item, the low-discomfort group felt significantly more strong about speaking after the workshops (*F* = 4.55, *p* = .034), but the feelings of the high-discomfort group did not significantly change over time. On the other hand, for the indicator “discomfort at work,” both groups felt more strong about speaking after the workshop, although the low-discomfort group felt only marginally more so. Neither of the negative emotions (irritable and nervous) changed over time.

#### Reconsideration of Research Career due to SC Skills

For the low-discomfort group, the level of reconsideration of career due to SC skills for both discomfort questions was around 2 (disagree) at baseline. However, six months post-workshop, the low-discomfort group shifted toward greater likelihood of reconsidering research careers (strongly disagree → disagree; *F*_judged_unfav_ = 5.92, *p* = .02; *F*_discomfort_at_work_ = 7.62, *p* = .006). For the high-discomfort group, scores for levels of reconsideration did not change significantly; this group maintained the same level of reconsidering. The scores in this group were around 3 (neither agree nor disagree) for both pre- and post-workshop surveys. Thus, the low-discomfort group became more likely to reconsider a research career due to language demands, while the high-discomfort group did not.

### Research Question 2

#### Reductions in Discomfort due to Language Variety

Table 4 shows estimated means (standard errors), *F* statistics (*p* values), and likelihood-ratio R^2^ for discomfort levels by language status. As mentioned in the Methods section, discomfort questions were asked in both pre- and post-workshop surveys beginning with the 2020 surveys (*n* = 88).

Regardless of language variety, mentees’ perception that they were “judged unfavorably” decreased significantly over time (*F*_st_en_ = 5.07, *p* = .027; *F*_o_en_ = 17.87, *p* < .001). Similarly, mentees in the “All Other” group felt less uncomfortable at work or school because of the way they speak at six months after the workshop (*F* = 7.04, *p* = .001), but the perceptions of standardized English–speaking mentees did not change: their scores were low at baseline and remained consistently low (*F* = .07, *p* = .79).

## Discussion

The current study sought to test whether an evidence-based SC mentoring intervention for mentors could result in changes to mentees’ outcomes related to language discomfort and the level of language discomfort felt by the mentees. We found that several career- and identity-associated attitudinal, affective, and cognitive indicators changed in desired directions for the high-discomfort group, including perception of the quality of communication with their mentor (marginal significance on the “discomfort in the lab” item); speaking productivity; and enthusiasm and confidence about speaking (feeling strong). Scores for the low discomfort group also increased for productivity of speaking, perception of their mentor’s impact on their speaking, and confidence and enthusiasm about speaking (marginally). Additionally, reconsideration of a research career due to the writing and speaking required was maintained at pre-intervention levels for the high-discomfort group but increased for the low-discomfort group.

Regarding changes in level of discomfort, perceptions of being judged unfavorably due to language variety and feelings of discomfort specifically at work or school due to language variety were reduced six-months-post-intervention, an important and novel finding.

Taking these findings together, we conclude that the SCOARE mentor-training workshop is effective in equipping STEM research mentors with skills that ameliorate several social-psychological and social-cognitive effects experienced by mentees who report feeling less comfortable in the research environment and/or judged unfavorably because of the way they speak and write English (or think they speak and write it).

Because a previous study found that SC productivity was a direct predictor of both SC self-efficacy and of intention to remain in a research career, gains in speaking productivity such as those experienced by the study mentee participants are important. The high-discomfort group of mentees reported increased quality of communication with their mentors, suggesting improvements in the mentor-mentee relationship itself. Finally, the high-discomfort group did not become more likely over time to reconsider a research career due to the communication demands involved, in contrast to the low-discomfort group, who did become more likely to reconsider due to the SC demands required. One possible explanation for this finding may be that respondents interpreted the question differently, with the high-discomfort group interpreting the phrase “due to the communication demands involved” to refer to the difficulty of SC, and with the low-discomfort group interpreting that question in terms of the effort involved. Another interpretation is related to general trends in diminished intention to pursue academic faculty research careers among doctoral and postdoctoral STEM trainees. “Intention to pursue a research career” is a distinct and more general construct, independent of concerns about SC, and has become more common over the last two decades (Fuhrmann et al., 2011; Gibbs et al., 2015). Our finding regarding reconsideration of career paths due to the SC demands involved might suggest a protective effect of the intervention for the high-discomfort group. Similarly, the low-discomfort group reported significantly greater impact of their mentor on their speaking tasks, while the high discomfort group did not show a statistically significant difference. Further investigation is needed to fully explain these patterns and their relationship to career intention.

Overall, these results support our previous findings regarding the role of language discomfort in the academic research setting and demonstrate that language discomfort is amenable to a workshop intervention for mentors that does not require learning specialized knowledge about writing instruction, language use, or sociolinguistics.

### Theoretical Considerations: Language Variety and Use

The SCOARE study followed emerging STEM researchers through their most advanced stages of training, when speaking and writing “like a scientist” becomes a key gatekeeping mechanism for academic career success. The SCOARE workshop intervention was designed to facilitate the socialization process by raising mentors’ awareness of language variation and its role in social and psychological processes. Our finding that mentees who experienced discomfort because of their language variety benefited differently when their mentors participated in an intervention sheds new light on the interactional processes that hinder or facilitate socialization into a particular identity and community. Importantly, we do not know whether the mentees in the study were using a nonstandardized variety of English in the research environment or in the presence of their mentors, although it is unlikely that many were, given the widespread use of standardized English at the postgraduate stage of education and the possibility of style-switching. Several faculty mentor participants who themselves were raised speaking a nonstandardized variety of English expressed in the workshop that they had always felt and were still feeling such discomfort, even as accomplished, tenured faculty These observations raise questions as to whether internalized linguistic stigma affects adults over their lifespan and despite acquiring additional varieties, as well as how prevalent internalized stigma might be, especially in high-register settings such as academia.

The current study extends the approach of linguistic models such as Gluszek and Dovidio’s (2010b) Stigma of Non-native Accents in Communication (SNAC) model and Clément and Kruidenier’s (1985) social-cognitive model of motivation to use a second language by examining language variety in general, not just accent, as in the SNAC model, and by including speakers of all varieties other than standardized English rather than a single population of L2 speakers. Although the experiences of nonnative speakers, regional dialect speakers, and ethnic sociolect speakers differ in important ways, our study suggests that they also have important commonalities within the context of gaining entry into a community of practice in which both high-register and discipline-specific language, spoken and written, are paramount for career success. Within the SCOARE study, there is no reference group based on a particular racial or ethnic identity; the reference group is based on sense of comfort or discomfort with home language variety in the community one seeks to join. This is a significant contribution of this study, as it facilitated study of the overall effects of language stigma and enabled the use of a generalized intervention that can benefit a spectrum of language backgrounds. With respect to interactional models, the SCOARE approach considers the experiences and knowledge of both partners in the communication (mentor and mentee) but does not examine real-time interactions per se, thereby allowing investigation of attitudes developed over time. Together with the use of a constructed (high vs. low discomfort) rather than demographically-based reference group, our focus on the social-psychological and behavioral outcomes for mentees in working with their mentors over time affords a more flexible view of communicative attitudes and processes.

### “Light-Touch” Social-Psychological Interventions

Yeager and Walton (2011) point out, in their review of social-psychological interventions targeting stereotype threat in education, that brief, simple exercises eliciting participants’ free expression of thoughts, feelings, and beliefs in and about school have been shown to have impressive and lasting behavioral benefits. These exercises foster “generative processes” in the cognition of the participants, such as a growth versus fixed mindset, values affirmation, or the reframing of negative experiences as environmental rather than personal. For example, Walton and Cohen (2011) found that a one-hour intervention in which students learned that feeling a lack of belonging in college was not due to their racial identity but was experienced by many freshmen led to a .24 increase in grade point average for Black students from their sophomore through senior years of college. Looking at language background, LaCosse et al. (2020) found that a brief belonging intervention among STEM-interested English L2 increased their anticipated sense of belonging and several academic outcomes. SCOARE is a longer intervention (6 hours) and is directed at mentors rather than mentees. Mentors learn to address language variety among their mentees not didactically, but through understanding important principles of language use and acquisition as well as education in general: that one does not acquire a new language variety or register overnight or at a writing workshop; that normalizing the experience of language discomfort is helpful; and that stimulating opportunities to speak spontaneously with colleagues on a frequent basis is preferable to waiting months for the mentee to produce a heavily edited publication or rehearsed presentation. Thus, SCOARE may be effective as a framing mechanism (Harackiewicz & Priniski, 2018) that enables mentors to reframe their view of the mentees’ perspective, capabilities, and needs. Initially, many faculty workshop participants see an either/or choice for mentees between use of nonstandardized and standardized varieties, and express concerns that encouraging mentees to acquire academic register and style would be detrimental to DEI efforts or to the mentee’s social identity, or, conversely, that if the mentor did not correct nonstandardized register and style, the mentee would not succeed in their career (per comments in workshop discussions and on workshop evaluations). One of the foundations of the SCOARE approach is the promotion of a “repertoire” perspective of language variety rather than an either/or, correct/incorrect perspective: when the mentor provides the necessary resources and support for mentees to acquire scientific register and style, postgraduate-level mentees can navigate language use according to their own purposes. Ultimately, SCOARE’s effect may be to help both mentor and mentee uncouple language variety from pejorative attributions about intellectual ability, attitude, or personality.

### Implications for DEI and Mentor Training

Examination of the construct of discomfort in the research environment due to language background and its association with nonstandardized varieties, including ethnic sociolects as well as L2 varieties, is a key contribution of this study and suggests future avenues for research. First, an individual’s language variety reflects important intersectionalities of race/ethnicity, geographic origin, and socioeconomic background, which can yield additional insights into studies of diversity and help us to better understand the needs and experiences of all individuals. Second, language variety is a fundamental component of identity, yet it is not an immutable characteristic in the way that heritage or physical features are. Thus, the inclusion of language variety as a factor in population-wide interventions may represent an important addition to the arsenal of methods to promote inclusivity. Finally, because the SCOARE mentor-training intervention does not rely on teaching specialized skills directly targeting the specifics of language use and variety, which would present a significant learning and application challenge, it is more amenable to scaled dissemination.

### Strengths and Limitations

The strengths of this study include its use of matched mentors and mentees and a pre/post design, the examination of broader social-psychological indicators related to language background and use rather than language attitudes only, and its interventional, evidence-based approach tested in the field. Some limitations to this study are also noted. Further research is needed to develop a validated scale to assess language-related discomfort more robustly and precisely and to consider possible additional correlates (e.g., avoidance behaviors associated with language-related discomfort). The instruments for eliciting self-reported language variety may benefit from further refinement. Mentor participants are a self-selected group; thus, only mentees of mentors concerned with developing mentee communication skills were included in the study.

### Future Directions

Analysis of mentor data will allow us to examine changes in mentors’ social-cognitive constructs and attitudes and whether they adopt new or different mentoring strategies after the workshop and provide more detailed insight into the mechanisms by which the workshop effects change. A detailed study of the overall mentee dataset will report on changes in social-cognitive career theoretical constructs not included here, such as self-efficacy for SC, outcome expectations for SC, science identity, and overall career intention among both mentees whose mentors attended the workshop and mentees whose mentors did not attend. More extensive investigation into between-group differences between sociolect, dialect, and L2 groups will likely yield additional insights: a greater proportion of regional dialect speakers belonged to the “judged unfavorably” group than to the “discomfort at work or school group,” while a greater proportion of ethnic sociolect and L2 speakers belonged to the “discomfort at work or school” group than the “judged unfavorably” group.

## Conclusion

This study found that effects of a workshop intervention that raises STEM mentors’ awareness of language variety and use and their relationship to career development cascaded to their doctoral and postdoctoral mentees over 6 months, reducing language discomfort; increasing verbal productivity, positive affect, and quality of communication with mentors; and limiting further reconsideration of research career plans of mentees reporting discomfort due to their language background. These outcomes have implications for the mediating role of language discomfort in studying the dynamics of language variety and suggest new possibilities for initiatives that raise awareness and broaden participation of linguistically diverse groups in research careers.

## Figures and Tables

**Table 1.** Mentee Sample Characteristics (n = 274)

Variable	Category	Frequency	Percentage
Gender	Female	177	65
	Male	95	35
	Other	2	0.7

Rank	Doctoral	200	73
	Postdoctoral	63	23
	Unknown	11	4

Discipline	Basic Science	150	55
	Population Science	18	7
	Clinical Science	65	24
	Other Science	41	15

Ethnicity	Hispanic	32	12
	Non-Hispanic	240	88
	Don’t know	2	0.7

Race	American Indian or Alaska Native (including all original peoples of the Americas)	1	0.4
	Asian or Asian American (including Indian subcontinent and Philippines)	77	28
	Black or African American (including Africa and the Caribbean)	19	7
	White or European American	156	57
	More than one race & Other	21	8

First generation	Yes	90	33
	No	182	66
	Don’t know	1	0.4

Disability	Yes	8	3
	No	266	97

Years working with mentor	Less than 1 year	76	28
	1 - less than 3 years	111	41
	3 or more years	87	32

Primary language	English	160	58
	Bilingual	39	14
	Other	75	27

**Table 2.** Frequency of Language-related ‘Discomfort’ by Home Language Variety

	Judged unfavorably because of “way I speak”		Discomfort at work or school because of “way I speak’	

Home language variety	Low	High	Low	High
Nonstandardized				
Ethnic sociolect (*n* = 37)	26 (70%)	11 (30%)	22 (60%)	15 (40%)
Regional dialect (*n* =28)	10 (36%)	18 (64%)	26 (93%)	2 (7%)
L2 (*n* = 75)	39 (52%)	36 (48%)	30 (40%)	45 (60%)
Other (*n* = 12)	5 (42%)	7 (58%)	10 (83%)	2 (17%)
Standardized (*n* = 122)	116 (95%)	6 (5%)	116 (95%)	6 (5%)

**Table 3.** Mixed-Effects Models for Changes in SC Outcomes

Judged unfavorably because of “way I speak”							
		
	Low discomfort			High discomfort			
		
	Mean estimates (standard errors)			Mean estimates (standard errors)			

SC outcomes	Pre-survey	6-month post-survey	*F* (*df*), *p*	Pre-survey	6-month post-survey	*F* (*df*), *p*	*R* ^2^
Quality of communication	4.07 (.09)	4.03 (.07)	.30, *p* = .58	3.84 (.11)	4.14 (.12)	**8.93, *p* = .003**	.03
Productivity in speaking tasks	3.90 (.10)	4.26 (.10)	**14.51, *p* < .001**	4.09 (.15)	4.5 (.16)	**7.41, *p* = .007**	.08
Impact of mentors on speaking tasks	3.77 (.05)	3.91 (.06)	**6.41, *p* = .012**	3.76 (.09)	3.86 (.09)	1.03, *p* =.31	.03
Affect, interested	3.64 (.08)	3.67 (.08)	.18, *p* = .67	3.64 (.12)	3.74 (.13)	.65, *p* = .42	.00
Affect, enthusiastic	3.29 (.09)	3.44 (.10)	3.32, *p* = .069	3.17 (.14)	3.48 (.15)	**4.95, *p* = .027**	.03
Affect, strong	2.99 (.09)	3.18 (.09)	**4.55, *p* = .034**	2.90 (.13)	3.14 (.14)	**2.97, *p* = .09**	.03
Affect, determined	3.15 (.08)	3.55 (.08)	.20, *p* = .65	3.42 (.13)	3.59 (.14)	1.54, *p* = .22	.01
Affect, irritated	1.47 (.06)	1.40 (.07)	1.33, *p* = .25	1.78 (.10)	1.88 (.11)	1.12, *p* = .29	.06
Affect, nervous	2.91 (.09)	2.72 (.09)	**4.12, *p* = .043**	3.14 (.14)	3.00 (.15)	.92, *p* = .34	.03
Reconsideration of career due to SC skills	2.10 (.09)	2.35 (.10)	**5.92, *p* = .02**	2.73 (.14)	2.83 (.15)	.34, *p* = .56	.09
Discomfort at work because of “the way I speak”							
	Low discomfort			High discomfort			
		
	Mean estimates (standard errors)			Mean estimates (standard errors)			
		
SC outcomes	Pre-survey	6-month post-survey	*F* (*df*), *p*	Pre-survey	6-month post-survey	*F* (*df*), *p*	*R* ^2^

Quality of communication	4.02 (.07)	4.03 (.07)	.02, *p* = .89	3.93 (.11)	4.14 (.12)	**3.75, *p* = .05**	.01
Productivity in speaking tasks	4.01 (.10)	4.39 (.10)	**17.28, *p* < .001**	3.80 (.16)	4.13 (.17)	**4.36, *p* = .038**	.09
Impact of mentors on speaking tasks	3.74 (.05)	3.87 (.06)	**5.95, *p* = .016**	3.86 (.09)	3.97 (.10)	1.43, *p* = .23	.03
Affect, interested	3.67 (.08)	3.71 (.08)	.22, *p* = .64	3.55 (.13)	3.64 (.14)	.54, *p* =.46	.01
Affect, enthusiastic	3.29 (.09)	3.44 (.09)	3.70, *p* = .056	3.17 (.15)	3.48 (.16)	**4.37, *p* = .038**	.03
Affect, strong	3.05 (.09)	3.22 (.09)	**3.88, *p* = .05**	2.73 (.14)	3.02 (.15)	**4.05, *p* = .045**	.04
Affect, determined	3.55 (.08)	3.57 (.08)	.10, *p* = .75	3.31 (.13)	3.53 (.14)	2.29,*p* = .13	.01
Affect, irritated	1.42 (.06)	1.39 (.06)	.28, *p* = .59	1.94 (.11)	1.94 (.11)	.00, *p* = .99	.09
Affect, nervous	2.86 (.09)	2.69 (.09)	3.47, *p* = .063	3.32 (.14)	3.12 (.15)	1.55, *p* = .21	.05
Reconsideration of career due to SC skills	1.94 (.08)	2.21 (.09)	**7.62, *p* = .006**	3.24 (.14)	3.21 (.15)	.02, *p* = .88	.26

*Note*. R^2^ = Likelihood ratio (LL). R^2^ is calculated as 1-exp(−2/n(LL_full-model_-LL_null-model_)).

**Table 4.** Mixed-Effects Models for Changes in Discomfort Levels

	Standardized English (st_en)			All other (ao_en)			
	
	Mean estimates (standard errors)			Mean estimates (standard errors)			

Outcomes	Pre-survey	6-month post-survey	*F* (*df*), *p*	Pre-survey	6-post-survey	*F* (*df*), *p*	Likelihood-ratio *R*^2^
Being judged unfavorably	1.39 (.14)	1.03 (.15)	5.07 *p* = .027	2.38 (.13)	1.81 (.13)	17.87 *p* < .001	.40
Discomfort at work	1.33 (.17)	1.28 (.17)	.07 *p* = .79	2.48 (.15)	2.08 (.15)	7.04, *p* = .001	.32

*Note*. Likelihood-ratio (LL) R^2^ is calculated as 1-exp(−2/n(LL_full model_-LL_null model_)).

## Data Availability

Data can be obtained from the corresponding author upon reasonable request.

## Appendix 1: SCOARE Workshop Agenda

**Table:**

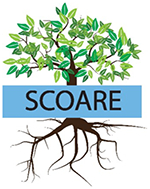

Session One	

**PART 1:**	**OVERVIEW**
	Welcome and introduction to the SCOARE workshop *Activity: Biggest SciComm Challenge (Discussion)*

**PART 2:**	**WHY IS COMMUNICATION SO IMPORTANT?**

	Language as thinking, identity, and communication Science versus scientific communication

	**5-MINUTE BREAK**

**PART 3:**	**EVIDENCE FOR ROLE OF SCICOMM IN RESEARCH TRAINING**

	Research background for the SCOARE workshop *Activity: Case Study – “Mentoring Scott” (Discussion)*
**PART 4:**	**UNDERSTANDING HOW LANGUAGE WORKS**

	Scientific style and language use
	**10-MINUTE BREAK**
	Three modes of language *Activity: Freewriting (Interactive)*
**PART 5:**	**YOUR ROLE AS A MENTOR**

	Engaged mentoring Setting expectations, structure, & accountability Acknowledgement strategies
**Session Two**	

**PART 5:**	**YOUR ROLE AS A MENTOR, CON’D**
	Introduction to giving feedback 5 helpful rules for feedback New ways to think about feedback *Activity: “Engaged’”Mentoring Discussion*
**PART 6:**	**SCAFFOLDING**

	Scaffolding speaking, presenting, and writing

	**5-MINUTE BREAK**

*Activity: Extended Exercise – Defining the Core of a Research Project Narrative*	

	**10-MINUTE BREAK**
**PART 7:**	**ENGAGEMENT AND PRODUCTIVITY**

	Engagement and productivity strategies *Activity: Your mentoring plan (Individual work, discussion)*
**PART 8:**	**LOOKING BACK AND MOVING FORWARD**

	Train to facilitate the SCOARE workshop Top 10 takeaways
**PART 9:**	**WORKSHOP EVALUATION**
	Workshop participant online evaluation**

** The participant evaluation is conducted by our external evaluator from the Learning Through Evaluation, Adaption, and Dissemination (LEAD) Center at UW-Madison. Workshop time is set aside for completion of the evaluation, and participants who complete it are eligible to receive 2 free books.

## Appendix 2. Language Variety Questions

Response options for the language variety item comprised 10 varieties including regional dialects and ethnic sociolects, most of which are found in general linguistic references such as the *Cambridge Encyclopedia of the English Language* (Crystal, 1995).

- Asian-influenced English
- Black English/African-American English [or Vernacular]
- Spanish-influenced English [“e.g., ‘Spanglish,’ ‘Tex-Mex,’ or other”])
- Noticeable Southern, Texas, or Cajun
- Appalachian or mid-Southern
- Noticeable Northeast urban [“e.g., Boston, New York, or other”]).
- California
- Noticeable upper Midwestern [“e.g., Chicago, Wisconsin, Minnesota, or other”]
- None
- Other (with option to write in)

The list of varieties was used in a previous study (Trachtenberg et al., 2018) and is not intended to refer to any specific, formally described varieties; multiple rounds of pretesting suggested that this list included the labels that were most readily understood by our participants, most of whom are unfamiliar with technical names of language varieties. Some of these labels, such as “Spanglish” and “Tex-Mex,” were suggested by pilot testers, and we found that using such names as paraphrases for more specialized terms was the most helpful for participants. “Asian-influenced English,” which we acknowledge as not recognized in the literature as a variety, was readily recognized by participants, despite the various and unrelated languages spoken by this group. All respondents were able to specify “Other” and/or a second variety if they so desired.
